# Assessing the effectiveness of two intervention methods for stony coral tissue loss disease on *Montastraea cavernosa*

**DOI:** 10.1038/s41598-021-86926-4

**Published:** 2021-04-21

**Authors:** Erin N. Shilling, Ian R. Combs, Joshua D. Voss

**Affiliations:** 1 Harbor Branch Oceanographic Institute, Florida Atlantic University, Fort Pierce, FL USA; 2 Present Address: Elizabeth Moore International Center for Coral Reef Research and Restoration, Mote Marine Laboratory, Summerland Key, FL USA

**Keywords:** Marine biology, Restoration ecology

## Abstract

Stony coral tissue loss disease (SCTLD) was first observed in Florida in 2014 and has since spread to multiple coral reefs across the wider Caribbean. The northern section of Florida’s Coral Reef has been heavily impacted by this outbreak, with some reefs experiencing as much as a 60% loss of living coral tissue area. We experimentally assessed the effectiveness of two intervention treatments on SCTLD-affected *Montastraea cavernosa* colonies in situ. Colonies were tagged and divided into three treatment groups: (1) chlorinated epoxy, (2) amoxicillin combined with CoreRx/Ocean Alchemists Base 2B, and (3) untreated controls. The experimental colonies were monitored periodically over 11 months to assess treatment effectiveness by tracking lesion development and overall disease status. The Base 2B plus amoxicillin treatment had a 95% success rate at healing individual disease lesions but did not necessarily prevent treated colonies from developing new lesions over time. Chlorinated epoxy treatments were not significantly different from untreated control colonies, suggesting that chlorinated epoxy treatments are an ineffective intervention technique for SCTLD. The results of this experiment expand management options during coral disease outbreaks and contribute to overall knowledge regarding coral health and disease.

## Introduction

Coral reefs face many threats, including, but not limited to, warming ocean temperatures, overfishing, increased nutrient and plastic pollution, hurricanes, ocean acidification, and disease outbreaks^1–6^. Coral diseases are complex, involving both pathogenic agents and coral immune responses. Some coral maladies are exacerbated by anthropogenic stressors as well as natural fluctuations in environmental conditions^7–12^. Many coral diseases are still poorly characterized, which has led to calls for increased research and intervention efforts to support adaptive management strategies^13–15^ particularly given the considerable impacts of diseases on coral reefs over the past five decades^4^. Two of the most notable events have affected multiple locations across the wider Caribbean^16–18^. White band disease caused a 98% reduction in benthic cover of reef-building *Acroporids* throughout some Caribbean reefs^19–22^. In 1995, a white plague type II outbreak affected 17 scleractinian species^23^, with some species such as *Dichocoenia stokesii* experiencing approximately 75% mortality in the Florida Keys^24,25^. Diseases continue to be a major threat to coral reef health, and continued ocean warming and eutrophication are likely to increase coral disease transmission, coral host susceptibility, and disease severity^2,5,16,18,26–28^.

Stony coral tissue loss disease (SCTLD) was first observed and described in 2014 in Miami-Dade County, and has since spread throughout the majority of the Florida Reef Tract and into multiple countries and territories in the Caribbean^29–31^. SCTLD is known to affect at least 20 stony coral species and appears to lack the seasonal or temporal fluctuation in incidence and prevalence commonly seen in other coral diseases, although relatively few environmental influence studies have been conducted on this newly described disease^31–33^. This combination of factors, along with rapid rates of tissue loss and high mortality, has devastated many coral reef communities^15,30,32,34^. Monitoring data in the southeast Florida region (Martin County through Miami-Dade County) suggests that there has been a 30% loss of live coral colony density and a 60% decrease in live coral tissue area in some regions since the start of the outbreak^15^. In the Mexican Caribbean, SCTLD prevalence increased from 0.5 to 25.9% in less than two years with an additional 12.9% of corals exhibiting recent mortality, presumably due to SCTLD^30^.

Preliminary ex situ trials tested different intervention methods directly on SCTLD-affected coral colonies, employing various iterations of physical barriers, trenching, and dosing with antibiotics or chlorinated agents^35,36^. These trials were conducted with five coral species: *Colpophyllia natans*, *D. stokesii*, *Meandrina meandrites*, *Montastraea cavernosa,* and *Pseudodiploria strigosa*. An ex situ trial of amoxicillin, gentamicin, paromomycin, and ampicillin as a treatment for SCTLD lesions on *Dendrogyra cylindrus* fragments had previously indicated that amoxicillin treatments resulted in the highest survival rate^37^. To administer amoxicillin to the corals more effectively and limit the amount of antibiotic leaching into the water column, a specially formulated silicone-based product, termed “Base 2B”, was developed (CoreRx/Ocean Alchemists LLC)^38^. Base 2B was designed to be combined with amoxicillin and applied in situ to disease lesions on stony corals, allowing for the release of amoxicillin over a 72-h period. Base 2B with amoxicillin increased the success rate of halting lesion progression by 38% as compared to using a shea butter mixture^35^. Therefore, Base 2B combined with amoxicillin was selected as the primary antibiotic intervention treatment for this study.

The chlorinated epoxy treatment was identified for preliminary in situ tests against SCTLD largely due to previously reported success with this method for treating black band disease in the Hawaiian archipelago^39^. Early trials with chlorinated epoxy as a treatment for SCTLD demonstrated some success (58% of individual lesions halted) during the first implementation in the northern section of Florida’s Coral Reef^40^.

This experimental in situ study was designed to assess field effectiveness of both amoxicillin plus Base 2B and chlorinated epoxy for treating SCTLD-affected corals in southeast Florida. Specifically, this study sought to determine 1) if these intervention methods effectively halt SCTLD lesion progression, 2) the duration of this efficacy, and 3) if either treatment provides colony-level resistance against future SCLTD events. Building upon preliminary ex situ and in situ trials, a controlled experimental design was implemented to evaluate chlorinated epoxy and Base 2B plus amoxicillin in comparison to untreated, naturally SCTLD-affected colonies over an 11-month period.

## Materials and methods

### Study site

**Figure 1:**
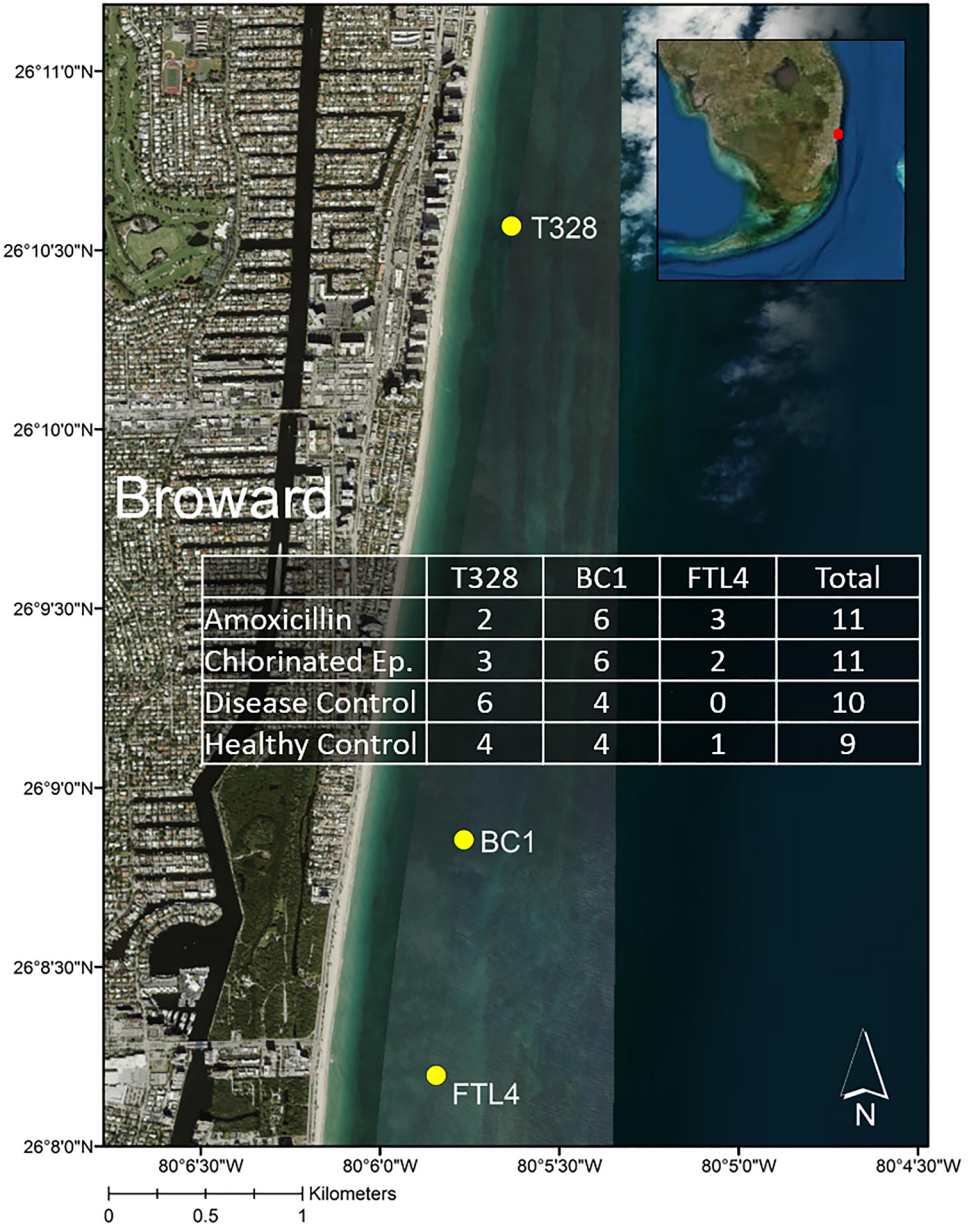
Satellite image of Broward County coast with sites utilized in this experiment denoted with yellow dots. The overlaid table lists sample sizes in each treatment group at each site, as well as treatment group totals across all sites. “Amoxicillin” refers to the Base 2B plus amoxicillin treatment, “Chlorinated Ep.” refers to the chlorinated epoxy treatment, “Disease Control” refers to the SCTLD-affected untreated colonies, and “Healthy Control” refers to healthy untreated controls. Map was created using ArcMap v10.1 software developed by ESRI (https ://www.esri.com).

This experiment was conducted approximately 2 km offshore from Lauderdale-by-the-Sea in Broward County, Florida (Fig. 1), at sites with a maximum depth of 10 m. SCTLD was first reported at these study sites in 2015^15^. Previous disease monitoring demonstrated that these sites had relatively high SCTLD prevalence and coral abundance at the time of this study as compared to nearby sites^41–43^.

### Study species

*Montastraea cavernosa* is a scleractinian coral found widely throughout the tropical western Atlantic, including within the regions currently affected by SCTLD^44^. This species was relatively abundant (making up more than 60% of live coral colonies > 10 cm in diameter)^43^ at the experimental sites following peak SCTLD prevalence in 2016, allowing for sufficient experimental replicates while maintaining a low likelihood of clones. *Montastraea cavernosa* is classified as “intermediately susceptible” to SCTLD, meaning it begins to show signs of the disease approximately one month after “highly susceptible” species, and typically experiences relatively slower rates of tissue loss which allows for longer-term fate-tracking of individuals^45^.

*Montastraea cavernosa* also exhibits a higher prevalence of SCTLD in this endemic study region as compared to other stony coral species, with *M. cavernosa* making up to 48–97% of all SCTLD-affected colonies observed at our sites prior to the commencement of this study^43^. Moreover, as a result of this outbreak, this species has experienced a significant reduction in density in southeast Florida, and preservation of *M. cavernosa* colonies is of particular importance due to its high abundance and role as a dominant reef builder in the northern section of Florida’s Coral Reef^15,46,47^. The ubiquity of *M. cavernosa* across the tropical western Atlantic also provides an opportunity for incorporating data and management implications from this research throughout the wider Caribbean.

### Experimental design

Experimental setup occurred over three days from 15 to 17 April 2019. Despite the relatively high abundance of *M. cavernosa*, the use of multiple sites (Fig. 1; site coordinates available in Supplementary Table S1) was necessary to achieve the desired number of replicates of SCTLD-affected and healthy coral colonies in the experiment. Coral colonies were tagged with numbered cattle tags across the three sites, all within ~ 16 km of each other. Tagged SCTLD-affected colonies used for a previous SCTLD-fate tracking study^41,42^ were supplemented with additional tagged, mapped, and monitored *M. cavernosa* SCTLD-affected and healthy colonies.

In total, 41 M*. cavernosa* colonies were tagged for this experiment: 32 SCTLD-affected colonies and 9 apparently healthy colonies. Due to the varying abundances of SCTLD-affected and healthy colonies at each of the three sites, an attempt was made to balance the intervention treatment groups across sites (Fig. 1). SCTLD-affected colonies were haphazardly divided into three groups: 1) Base 2B plus amoxicillin treatment; 2) chlorinated epoxy treatment; 3) untreated control group. The apparently healthy colonies were used as controls to assess incidence of SCTLD in the natural population and potential non-SCTLD-associated mortality in the region. The term “apparently healthy” is used as colonies with SCTLD have been observed to quiesce and not show signs of SCTLD for months, only to become active again later. Only visibly healthy corals that also showed no signs of recent tissue loss (characterized by bare or recently algae-colonized skeleton) were selected as healthy controls.

### Intervention treatments

**Figure 2:**
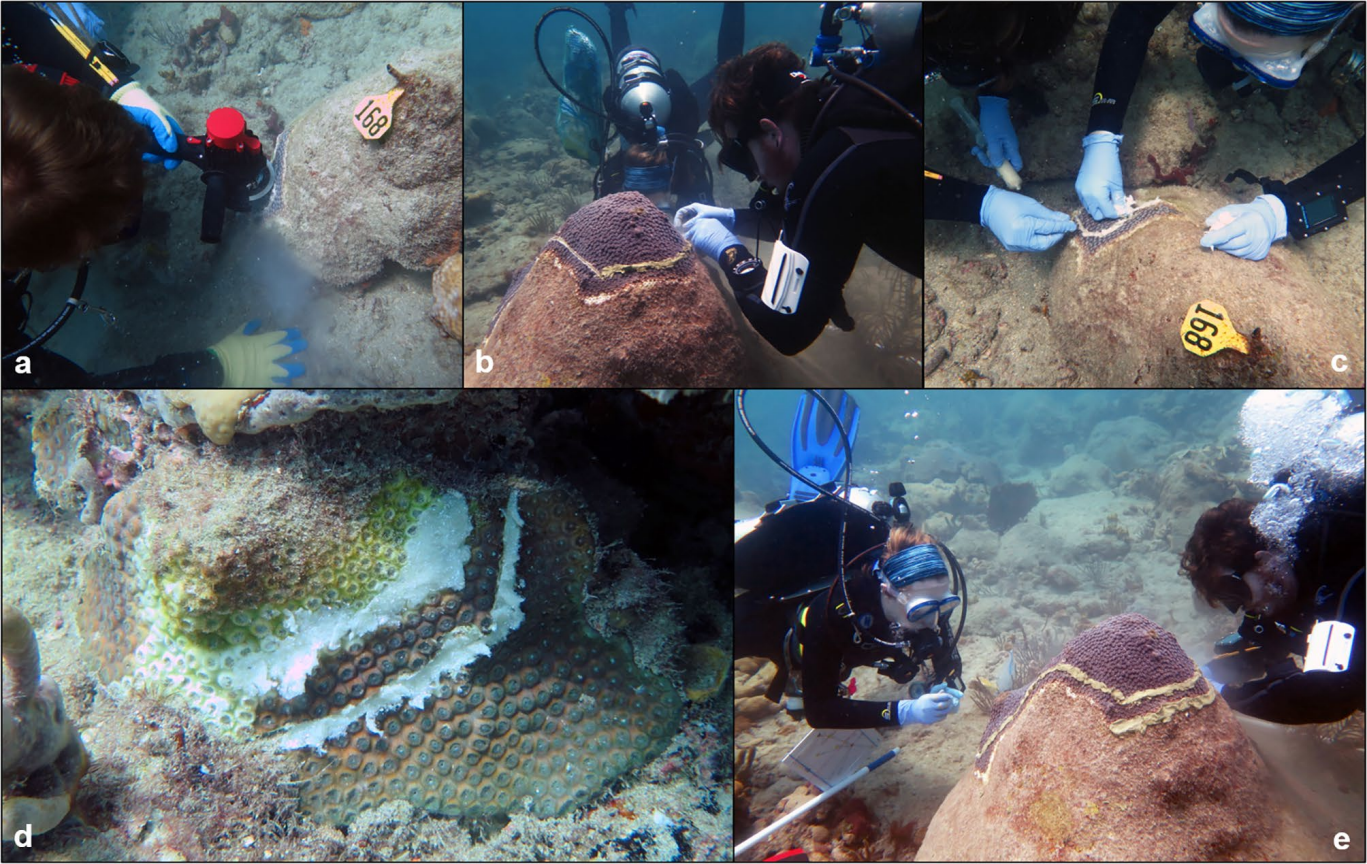
Step-by-step process of treating SCTLD-affected coral colonies in situ. (**a**) Diver creating a trench around the SCTLD lesion using an angle grinder. (**b**) Filling a trench with the chlorinated epoxy treatment. (**c**) Filling a trench with the Base 2B plus amoxicillin mixture. (**d**) A SCTLD-affected coral colony that has been treated with the Base 2B plus amoxicillin mixture. (**e**) A SCTLD-affected coral colony partially treated with the chlorinated epoxy.

All intervention treatments were initiated with the creation of trenches around all SCTLD lesions present on the colony. Trenches were cut approximately 1 cm deep × 1 cm wide and ~ 5 cm away from the disease margin using a Nemo underwater angle grinder (Fig. 2a), creating a buffer of apparently healthy tissue to prevent potential SCTLD progression through sub-surface tissues^45^.

The CoreRx/Ocean Alchemists Base 2B plus amoxicillin treatment was created by combining Base 2B with powdered amoxicillin trihydrate (Phytotech Labs) in a 10:1 mass ratio mixture. Both components were kept on ice until combined on the research vessels immediately before dives, as recommended by Ocean Alchemists LLC. Underwater, the mixture was both packed into the trenches and spread over the entirety of each SCTLD lesion (Fig. 2c,d).

The chlorinated epoxy treatment included Poolife Turboshock calcium hypochlorite-based powder (77% Ca(OCl)_2_) and ZSPAR A-788 Splash Zone two-part epoxy combined in an approximate volumetric ratio of 3:10 mL calcium hypochlorite powder:part A epoxy (Fig. 2b,e). This mixture was subsequently combined and thoroughly mixed with the part B epoxy in equal proportions underwater immediately before application to the colony’s trenches and lesions as described above. Z-Spar is designed to set within two hours after application and be completely hardened within 6–8 h. Therefore, it is estimated that release of calcium hypochlorite from the epoxy occurred for a maximum 8-h period following application.

Scaled photographs were taken of each experimental *M. cavernosa* colony immediately before and after the intervention treatments were applied. Additionally, videos were recorded before interventions for 3D model generation to measure initial colony surface area of all SCTLD-affected experimental colonies.

### Monitoring of experimental colonies

**Figure 3:**
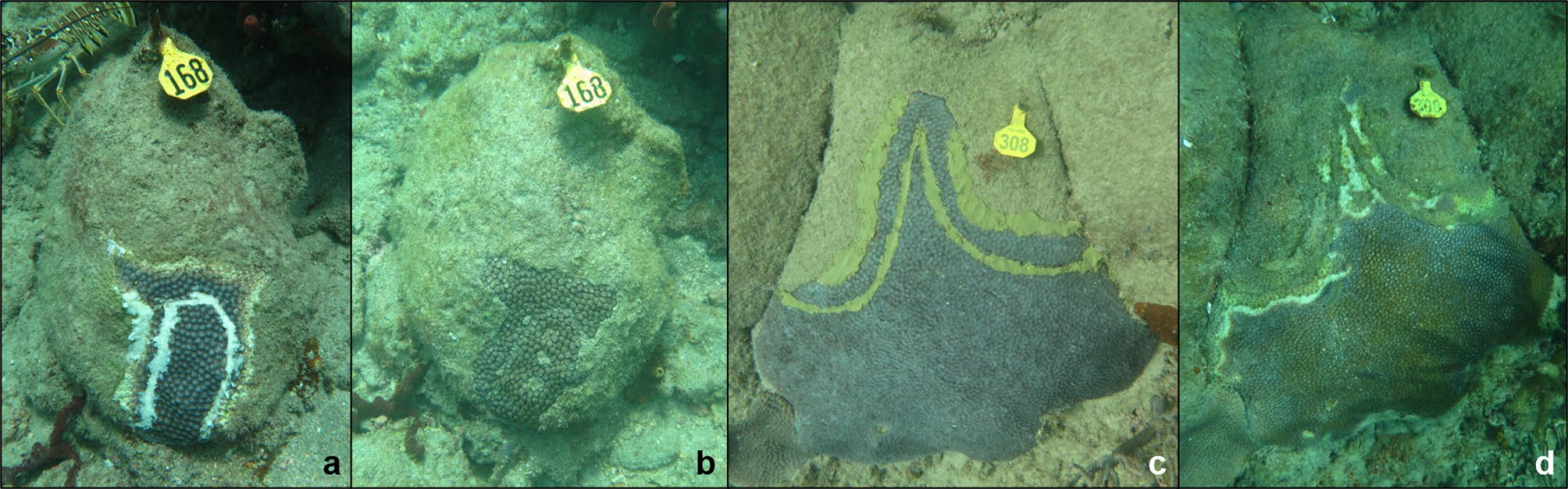
Examples of treated *Montastraea cavernosa* coral colonies with and without tissue remaining between trenched areas and the initial disease margin. 3a & 3b show a Base 2B plus amoxicillin treated colony (**a**) immediately after treatment application and (**b**) 46 weeks after treatment, with almost all initial coral tissue remaining. 3c & 3d show a representative chlorinated epoxy treated *M. cavernosa* colony (**c**) immediately after treatment and (**d**) nine weeks after treatment, with the lesions progressing across the trench.

Experimental coral colonies were revisited six times over an 11-month period following the application of intervention treatments. After the initial setup, revisits for monitoring occurred at approximately three, five, nine, fourteen, twenty-three, and forty-six weeks. At each monitoring time point, SCTLD activity, number of lesions, and tissue presence or absence between the initially trenched area and the disease margin (Fig. 3) were recorded, and scaled photographs were taken for each experimental colony. Colony SCTLD status was categorized as one of the following: a) “diseased”: visible lesions and active tissue loss occurring on the colony, b) “quiesced”: the colony was previously diseased but at the time of inspection had no visible lesions or active tissue loss, c) “dead”: no live tissue, healthy or diseased, was remaining from the original colony, or d) “apparently healthy”: this only pertained to the healthy control colonies, as they were not currently diseased and had never been observed with disease signs. In addition to tracking the total number and new lesions on a colony, the status of each individual lesion that was present at the beginning of the experiment (on both treated and untreated colonies) was monitored. Lesions exhibiting signs consistent with SCTLD, including active tissue loss and/or paling or bleaching of coral tissue at the lesion margin^45^, were classified as an active disease lesion. The majority of lesions at all monitoring time points (> 95%) were consistent with what has been described as “subacute” tissue loss and included the characteristic band of bleached tissue^32^. The remainder (< 5%) showed “acute” tissue loss without paling or bleached tissue. Due to this small difference in lesion types present, no analysis of any potential relationships between lesion type and treatment groups was pursued.

### 3D model generation and analysis

Videos of the colonies were recorded immediately before intervention and 3D models were later generated using methods described in Combs et al.^41,42^. Briefly, stills were extracted from the videos using the software FFmpeg, and then used to generate the models through a four-step process in the software program Agisoft Metashape (Version 1.5.2, Agisoft LLC). All tracing and quantifying of tissue areas from coral colony models was conducted in the application software Rhinoceros 3D (McNeel & Associates). Models and tissue areas were only generated for initially SCTLD-affected coral colonies in this experiment. Therefore, since none of the healthy controls showed any signs of SCTLD or tissue loss throughout the course of the experiment, none of them were included in this 3D modelling analysis or any subsequent SCTLD lesion analyses.

### Statistical analyses

All figures and statistical analyses were generated in the *R* statistical environment, version 3.5.2^48^. Figures were generated using the packages *ggplot2* and *Rmisc*^49,50^. Shapiro–Wilk tests indicated the datasets were not normally distributed, and transformations to normalize the data were unsuccessful. For all initial lesions present, total, and new lesion developed analyses, the mean number of lesions on each colony were used. Kruskal–Wallis tests were conducted to assess (1) if initial colony surface area was different between treatment groups, site, or had any influence on disease status at 46 weeks, (2) if site had an influence on the total number of lesions or the cumulative number of new lesions on a colony at 46 weeks, (3) if the colonies across treatment groups had different numbers of new lesions at each time point, or cumulative new lesions by 46 weeks, and (4) if there were any differences in the initial number of lesions present on SCTLD-affected colonies across sites or treatment groups at the start of the experiment. Fisher’s tests were run to determine if site had an influence on the disease status of a colony at any time point. Subsequent tests were run grouping colonies from all sites by treatment group. For all lesion status-based analyses, lesions were assessed independently of the colonies, meaning in several cases multiple lesions were assessed independently that existed on a single colony.

Fisher’s exact tests & pairwise Fisher’s tests, using the package *rstatix,* were used to detect any potential relationship between treatment group and colony disease status at each monitoring time point, or treatment and the initially treated SCTLD lesion status at 46 weeks^51^. Both colony disease status and treated lesion status were analyzed independently so that the treatment’s effectiveness at halting individual lesions could be assessed while also determining if a treatment had any impact on the colony as a whole. Kruskal–Wallis tests were run to determine if the mean initial number of lesions present on a colony had any influence on its disease status at any of the six follow-up monitoring time points, first with all SCTLD-affected colonies grouped, and then blocked by treatment group. A Fisher’s exact test was used to look for any difference between the likelihood of live tissue remaining between the trenched areas and the initial disease margin chlorinated epoxy and amoxicillin treated colonies at 46 weeks. Spearman’s rank correlation tests were run to identify any associations between time and the development of new lesions or total lesions present on a colony across treatment groups. Spearman’s rank correlation tests were also run to look for associations between initial colony surface area and the initial number of SCTLD lesions present, as well as new lesions developed. Dunn’s tests were run as post hoc analyses for all significant Kruskal–Wallis tests, using the package *FSA*^52^. All *p*-values from pairwise comparisons were adjusted using the Bonferroni correction method, and the alpha level for all statistical tests was 0.05.

## Results

### No significant site or colony effects

Kruskal–Wallis tests indicated that site had no significant effect on cumulative new lesion development over 46 weeks (11 months) or the total lesions present on a colony at 46 weeks (Kruskal–Wallis tests, all *p* > 0.05, Supplementary Table S2). Site also had no influence on the SCTLD status of a colony at any time point (Fisher’s exact tests, all *p* > 0.05, Supplementary Table S3). There was also no significant difference between the initial numbers of lesions on experimental colonies between sites or treatment groups (Kruskal–Wallis tests, all *p* > 0.05, Supplementary Table S4). The initial number of SCTLD lesions present on a colony at the beginning of the experiment had no influence on its SCTLD status at any follow-up monitoring time point, even when blocked by treatment group (Kruskal–Wallis tests, all *p* > 0.05, Supplementary Table S5). Three experimental colonies (one from each SCTLD-affected treatment group) were excluded from the initial colony surface area analyses because the generated models were poor quality and could not be measured accurately. Mean initial colony surface area of all SCTLD-affected experimental corals was 2,743.01 ± 206.62 cm^2^ SD with a range of 91.17–10,174.48 cm^2^. Mean initial colony surface area did not differ between sites or treatment groups (Kruskal–Wallis tests, both* p* > 0.05, Supplementary Table S6). Initial colony surface area had no effect on the disease status of an initially SCTLD-affected coral at 46 weeks, even when blocked by treatment group (Kruskal–Wallis tests, all *p* > 0.05, Supplementary Table S6).

### Impact of treatment on lesion and colony SCTLD status

**Figure 4:**
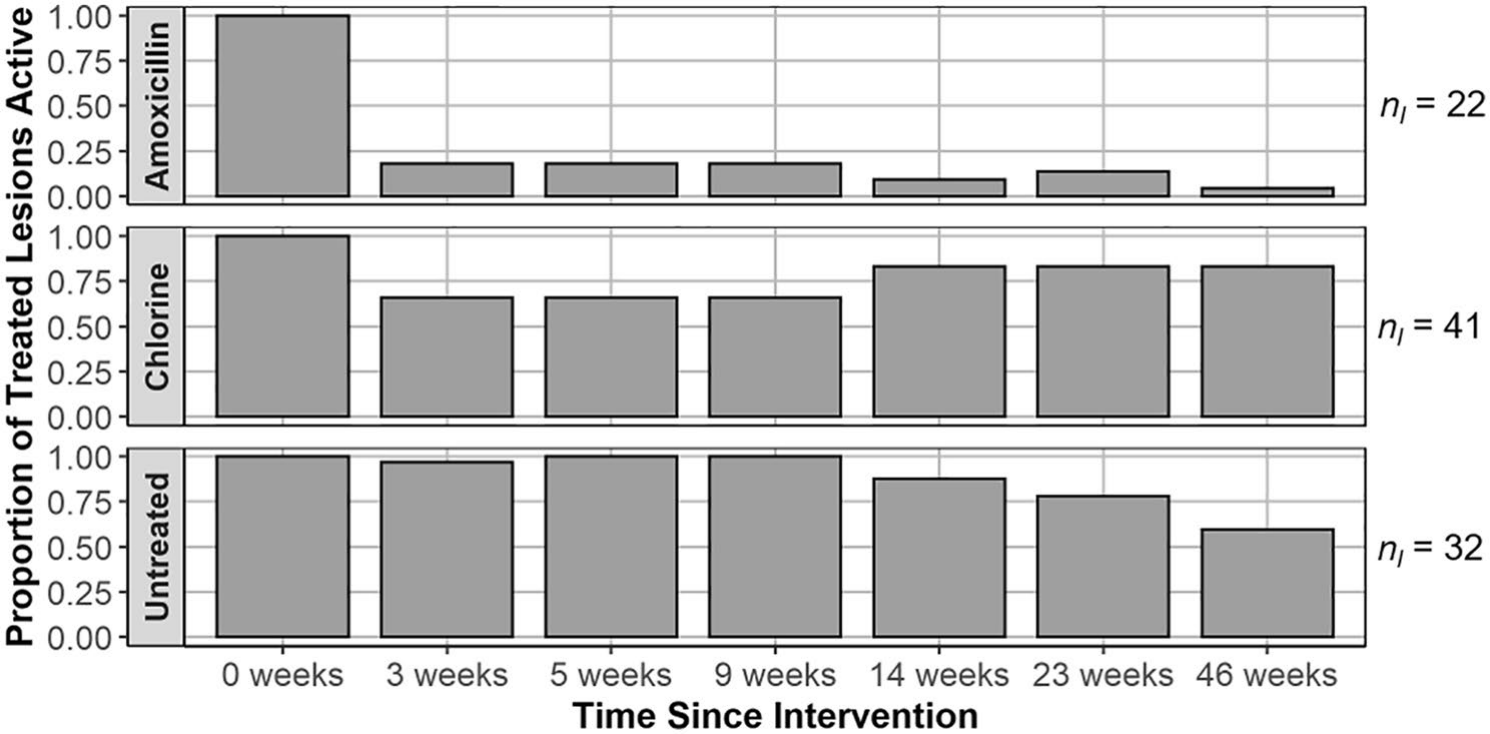
Proportion of initially treated disease lesions active on colonies by treatment group at each monitoring event, with *n*_*l*_ indicating total number of lesions present across all colonies in the treatment group. Amoxicillin refers to the Base 2B plus amoxicillin treatment, chlorine refers to the chlorinated epoxy treatment, and untreated refers to the SCTLD-affected controls.

At 46 weeks, the amoxicillin treated lesions had the highest quiescence rate at 95%. This success of amoxicillin treated lesions was significantly higher than the quiescence rates for untreated lesions and chlorinated epoxy treated lesions at this time point (Fisher’s exact test, *n* = 95, *p* < 0.001; pairwise Fisher’s test, all *p* < 0.001; Fig. 4). There was no significant difference detected between the quiescence rates of chlorinated epoxy and untreated lesions at 46 weeks. Coral colonies that were in the Base 2B plus amoxicillin treatment group were more likely to have tissue remaining between the trenched barriers and the initial disease margin (71%, *n* = 14) when compared to the chlorinated epoxy treated coral colonies (6%, *n* = 18) 46 weeks after treatment (Fisher’s exact test, *p* < 0.001).

**Figure 5:**
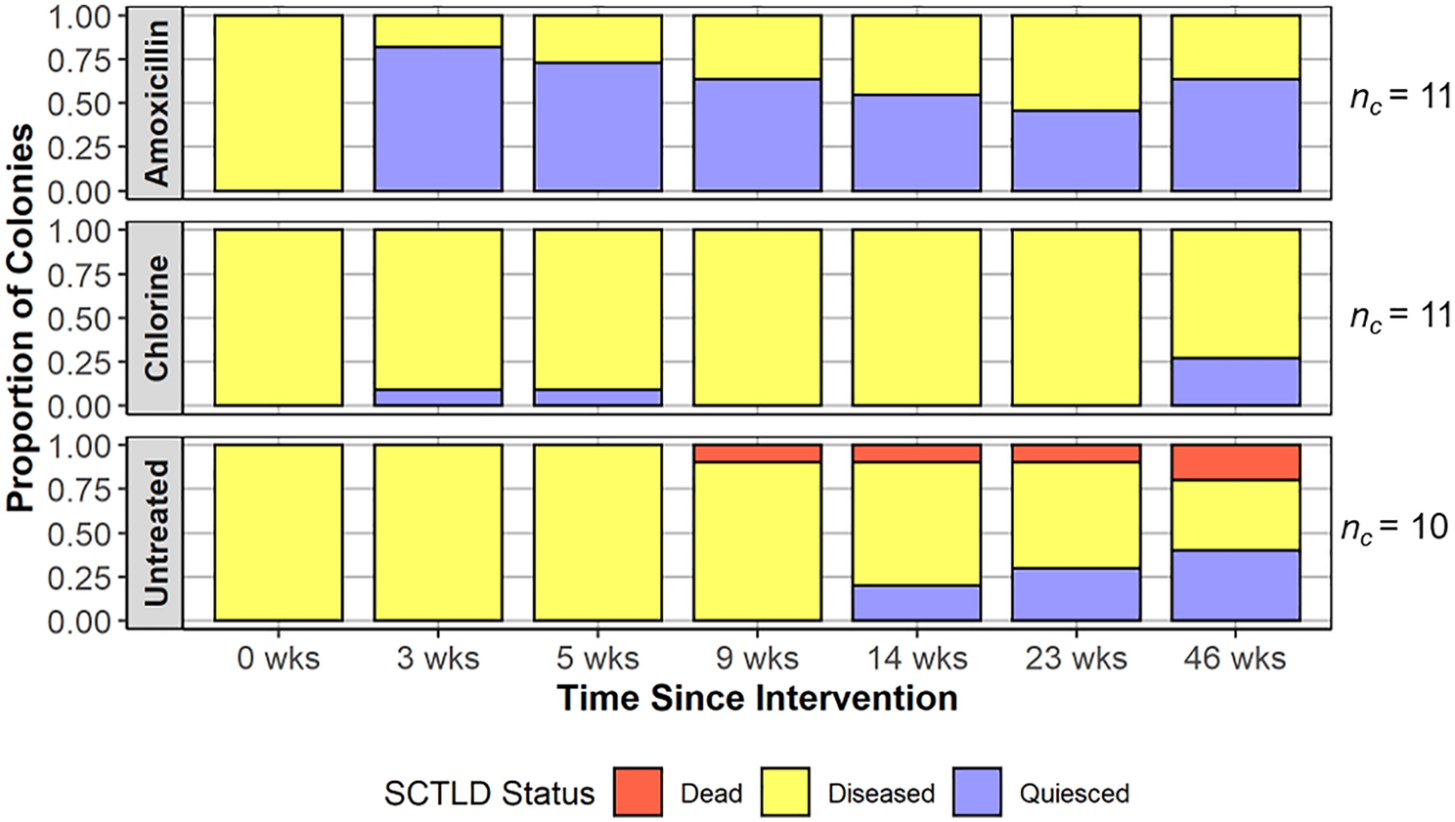
Disease status of colonies by treatment group at each time point shown in proportions of total, with *n*_*c*_ representing the total number of colonies in the treatment group. Amoxicillin refers to the Base 2B plus amoxicillin treatment, chlorine refers to the chlorinated epoxy treatment, and untreated refers to the SCTLD-affected controls.

Treatment significantly influenced the SCTLD status of a colony until the 46-week time point (Fisher’s tests; Table 1; Fig. 5). From the first monitoring at three weeks to the third monitoring at nine weeks, the amoxicillin treated colonies were more likely to be completely quiesced than the chlorinated epoxy treated or untreated colonies (Fisher’s pairwise tests, Table 1). At the fourth monitoring at 14 weeks, the amoxicillin treated were only more likely to be quiesced than chlorinated epoxy treated colonies. At the fifth monitoring event at 23 weeks, the global Fisher test indicated a borderline significant influence of treatment, however after Bonferroni corrections on *p*-values from the pairwise tests, none were significant (Table 1).

**Table 1:** Fisher’s exact tests and pairwise Fisher’s exact test comparing SCTLD status between treatment groups at each time point.

	Treatment influence on colony SCTLD status			
	Global test	A vs. CL	A vs. C	CL vs. C
3 weeks	** < 0.001**	**0.006**	** < 0.001**	1
5 weeks	** < 0.001**	**0.023**	**0.003**	1
9 weeks	** < 0.001**	**0.012**	**0.012**	1
14 weeks	**0.008**	**0.037**	0.549	0.642
23 weeks	**0.043**	0.105	1	0.271
46 weeks	0.244	NA	NA	NA

Specifically, the *p* values that are significant for each pairwise comparison represent a difference detected between quiesced vs. not quiesced (dead or diseased) at that time point. A = Base 2B plus amoxicillin treatment (n = 11), CL = chlorinated epoxy treatment (n = 11), and C = untreated SCTLD-affected controls (n = 10). Significant p-values are bolded, NAs represent lack of pairwise comparisons due to insignificant global test.

### Development of lesions on colonies throughout experiment

**Figure 6:**
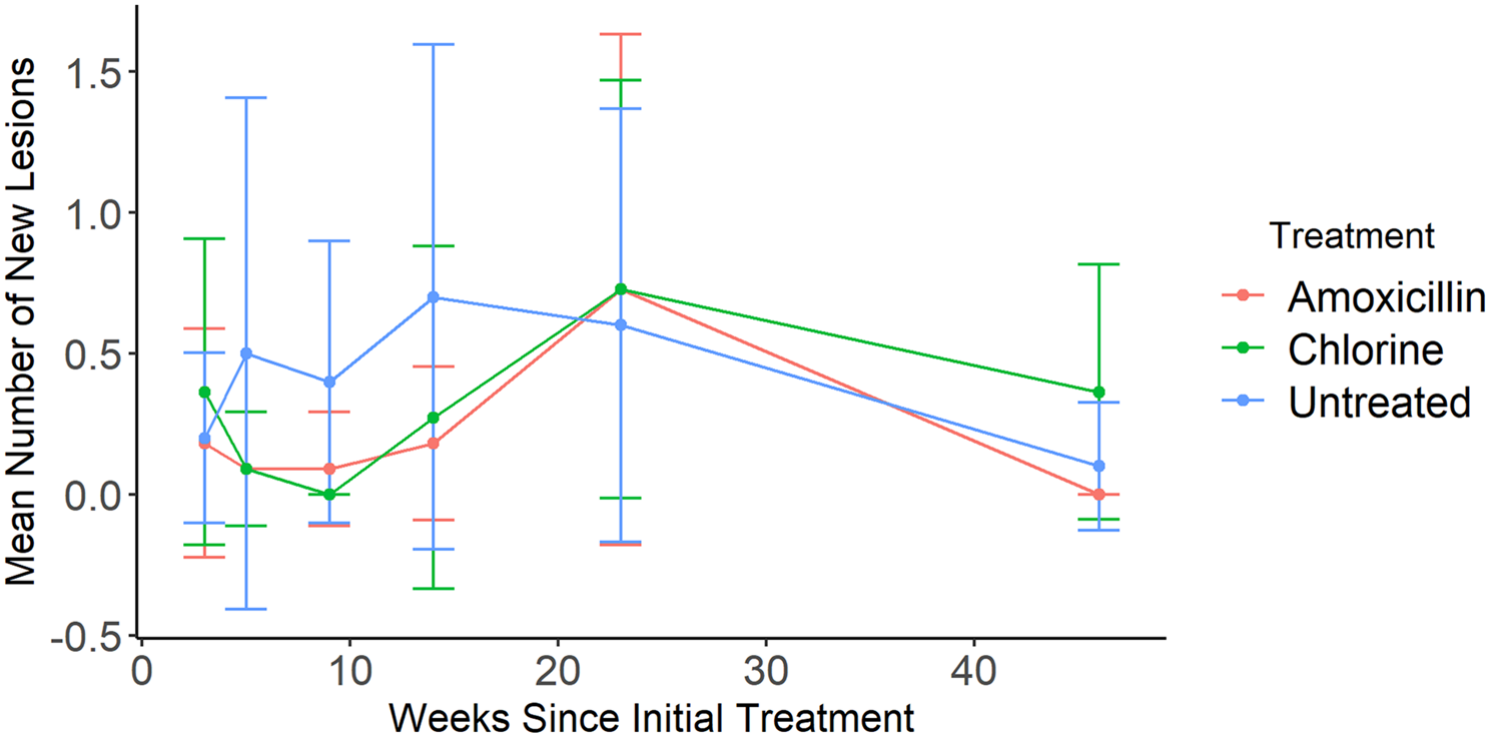
Mean number of new SCTLD lesions developed on a colony since the previous monitoring event, beginning at 3 weeks and ending at 46 weeks, grouped by treatment. Amoxicillin refers to the Base 2B plus amoxicillin treatment, chlorine refers to the chlorinated epoxy treatment, and untreated refers to the SCTLD-affected controls. Bars represent 95% confidence interval.

There was weak or no correlation detected between either the number of new lesions developed on colonies and time (Fig. 6), or total lesions present on colonies and time, even when blocked by treatment group (Spearman’s rank correlation, *p* > 0.05 in all cases, Supplementary Table S7). There was a significant positive correlation between initial colony surface area and the initial number of lesions present (Spearman’s rank correlation, *n* = 30, ρ = 0.469, *p* < 0.001). However, the results from the correlation analyses are skewed by one outlier, specifically the largest colony which had almost four times as many lesions as any of the other colonies. With this individual excluded, the correlation remained significant but showed a weaker relationship (Spearman’s rank correlation, *n* = 29, ρ = 0.405, *p* < 0.001). There was also a significant positive relationship between initial colony surface area and the number of new lesions accumulated over the course of the entire experiment (Spearman’s rank correlation, *n* = 28, ρ = 0.40, *p* = 0.035). There was no influence of treatment on new lesion development. This was confirmed by comparing new lesions present on a colony between each time point as well as new lesions accumulated on a colony over the course of the entire experiment (Kruskal–Wallis test, all *p* > 0.05, Supplementary Table S8).

## Discussion

In this experiment the Base 2B plus amoxicillin treatment was significantly more effective at treating individual SCTLD lesions on *M. cavernosa* colonies than the chlorinated epoxy or leaving the lesions untreated. This study supports and reinforces previous reports of successful antibiotic application for the treatment or diagnosis of coral wounds and disease. Sweet et al.^53^ were able to completely arrest white band disease lesions with ampicillin and paromomycin on *Acropora cervicornis* fragments in a laboratory experiment. Aeby and colleagues^32^ halted SCTLD lesion progression on three coral species by dosing the water with nalidixic acid or a combination of amoxicillin and kanamycin as a means of potentially diagnosing the etiological agents of the disease. Another study also utilized the Base 2B plus amoxicillin treatment on SCTLD-affected corals in situ, albeit without the use of trenches. They observed a similar success rate of 89% observed on *M. cavernosa* lesions that were treated and tracked over a two month period, along with a 67–91% success rate on four other treated species^54^. While successful demonstration of these techniques is an important development in coral disease research, the issue remains of the feasibility of these methods for in situ use at a larger scale. This study and Neely et al. 2020^54^ demonstrate that the Base 2B plus amoxicillin treatment is not only effective at treating SCTLD lesions, but that it can be used relatively easily by SCUBA divers. The Base 2B plus amoxicillin treatment did not remain attached to all portions of each lesion or trench due to occasional difficulty with adherence to the coral tissue; however, this did not appear to impede the antibiotic treatment’s effectiveness.

The chlorinated epoxy treatment tested in this experiment was ineffective for SCTLD lesions on *M. cavernosa.* SCTLD lesions treated with chlorinated epoxy were no more likely to quiesce than lesions left untreated. The short-term release of the calcium hypochlorite from the epoxy treatment during curing time may be insufficient to kill SCTLD pathogens. Similar chlorinated treatments have demonstrated efficacy against black band disease (BBD) in corals^39^. BBD is caused by a suite of bacterial pathogens^55–58^; whereas the pathogenic agent(s) or physiological responses associated with SCTLD remain unknown and may be unresponsive to this type of treatment. In addition to applying chlorinated epoxy, Aeby et al.^39^ also mechanically removed the BBD bacterial mat prior to chlorinated epoxy treatment, similar to Hudson’s^59^ methods using an underwater aspirator. This could have been a contributing factor to the success of chlorinated epoxy on BBD vs. SCTLD-affected colonies. These comparative differences highlight that both the intervention agent (antibiotic, antiseptic, etc.) and the application media (epoxy, shea butter, silicone base, etc.) must be optimized and tested for each coral disease being treated. Alternative methods using slower curing application media designed to release a chlorinated agent more slowly could also be tested to see if they improve success rates for a chlorinated treatment for SCTLD.

In this experiment, living coral tissue between the trenches and initial disease margin was more often preserved on Base 2B plus amoxicillin treated colonies. When the chlorinated epoxy applied directly over the SCTLD lesion did not prevent progression, the trenches also did not prevent SCTLD from continuing to progress across the coral colonies. Given the evidence that SCTLD is likely waterborne and is being transported by currents^31,32,45^, the utility of the trench is uncertain. Randall et al.^60^ tested trenching alone to treat yellow band disease on *Orbicella faveolata* and showed high initial success of halting lesion progression, but after 19 months reported a success of only 10%. Trenching success at halting SCTLD lesion progression on *O. faveolata* has been reported as 48.9–68.0% at two to three months post-treatment^40,61,62^. Given this generally low success of trenches halting disease progression on chlorinated epoxy treated colonies, and the high frequency of tissue preservation between the margin and trench observed in this study, we suggest that in instances where time and effort underwater are constrained, application of Base 2B plus amoxicillin to more SCTLD-affected colonies should be prioritized over supplementing the antibiotic treatments with trenching. However, a controlled experiment comparing trenching and Base 2B plus amoxicillin treatments versus Base 2B plus amoxicillin alone is recommended to further optimize effective and efficient treatment methods and evaluate potential negative consequences of trenching, as well as evaluate the relative risks and trade-offs associated with mechanical trenching.

In the broader context of characterizing of SCTLD, these observations of successful antibiotic applications and only minor increased efficacy through trenching suggests that the causative agent of SCTLD is likely bacterial but could also be a viral agent or a generalized physiological response that is then colonized by opportunistic bacteria. Further histological analyses are needed to examine the effects of Base 2B plus amoxicillin on the lesion and near-lesion. Likewise, the consequences of antibiotic applications to SCTLD-affected colonies need to be quantified, particularly the potential effects on coral mucus microbial assemblages and possible changes in antibiotic resistance.

Finally, while Base 2B plus amoxicillin demonstrated high efficacy against SCTLD lesions in this experiment, the antibiotic treatment does not prevent the colony from developing new SCTLD lesions in other locations on a colony over time. This is consistent with other in situ and ex situ trials previously conducted on this disease, and suggests the need for repeated antibiotic treatments to effectively halt SCTLD impacts on a colony^32,54^. Success in treating SCTLD with antibiotics may benefit from using approaches typically successful against bacterial infections in humans, for example using a strong initial dose of antibiotics followed by a regimen of smaller supplementary doses over time^63^. The appearance of new lesions in this study after all Base 2B plus amoxicillin treated lesions had healed suggests three potential scenarios: 1) the causative agent of SCTLD is still present in the environment and is re-infecting quiesced colonies, 2) the duration and dose of Base 2B plus amoxicillin is sufficient to arrest SCTLD at treated lesions, but insufficient for eliminating SCTLD pathogens from other areas of the coral colony, or 3) the coral immune system is compromised from this original SCTLD affliction and opportunistic bacteria are able to cause secondary infections observed as lesions.

Over the course of 11 months of monitoring, which began in late spring and ended in late winter, no clear trends were observed in SCTLD lesion development which could be linked to temperature or seasonality, either on treated or untreated colonies. The observations of new lesion development in this study, however, do suggest that follow-up evaluation for treatment of any new lesions that have developed should occur approximately between two and three months after initial antibiotic application on SCTLD-affected *M. cavernosa* colonies. At 14 weeks amoxicillin treatments were no longer significantly different than untreated controls in terms of SCTLD lesion quiescence (Table 1, Fig. 5). Subsequent monitoring should likely be conducted every two months onward to treat new lesions as they develop. These timelines may apply to other “moderately susceptible” coral species as well, given that they are in part classified by disease progression rate, but further species-specific trials should be conducted to confirm. “Highly susceptible” species may need even more frequent follow-ups. Lesion-level success should be determined by the halting of the treated lesion after one treatment, but colony-level success, which is more important in the long term, should be determined by the prevention of new lesion development on the treated colony. Additional empirical research and controlled studies are needed to determine if there are regional, temporal, and/or species-specific influences on intervention treatment success. Nonetheless, the results of this study demonstrate that Base 2B plus amoxicillin treatments can be effective against SCTLD and provide a viable management option for mitigating the impacts of SCTLD.

The success of Base 2B plus amoxicillin treatments is encouraging in the face of a disease outbreak that is continuing to devastate Caribbean coral reefs. However, potential secondary impacts of amoxicillin treatments on SCTLD-affected corals remain uncharacterized. We recommend that future research efforts focus on assessing the potential unintended consequences of antibiotic treatments on corals, their microbial communities (including Symbiodinaceae), and neighboring organisms. Additionally, further efforts are needed to optimize dosing and delivery methods for antibiotic treatments on SCTLD-affected corals and scale up intervention treatments effectively.

## Supplementary Information

Supplementary Information

## Data Availability

Statistical analysis scripts and documentation, as well as all datasets used to conduct these analyses are available on GitHub (https://github.com/erin-shilling/SCTLDintervention_ScientificReports). The full 3D model generation protocol is also available on GitHub (https://github.com/icombs2017/analysisOf3dModels).
