# Supplementary material for: Assessing the effectiveness of two intervention methods for stony coral tissue loss disease on Montastraea cavernosa
Source: Sci Rep. 2021 Apr 21;11:8566. doi: 10.1038/s41598-021-86926-4 (PMC8060409; doi:10.1038/s41598-021-86926-4)
Supplement: Supplementary file 1 — Supplementary Information [file 41598_2021_86926_MOESM1_ESM.pdf]

Supplemental Information

Assessing the effectiveness of two intervention methods for stony coral tissue loss disease on *Montastraea cavernosa*

*Erin N. Shilling[1], Ian R. Combs[1,2], *Joshua D. Voss[1]

[1]Harbor Branch Oceanographic Institute, Florida Atlantic University, Fort Pierce, Florida, United States of America

[2]Current address: Elizabeth Moore International Center for Coral Reef Research & Restoration, Mote Marine Laboratory, Summerland Key, Florida, United States of America

Table S1. GPS coordinates of study sites.

Site Name	Latitude	Longitude
T328	26˚ 10.567' N	80˚ 05.633' W
BC1	26˚ 08.855' N	80° 05.633' W
FTL4	26˚ 08.197' N	80˚ 05.843' W

Table S2. Results of Kruskal-Wallis tests run to assess if site had any influence on cumulative new lesions and total SCTLD lesions developed by 46 weeks.

Test	Comparison	*n*	*H*	*p*-value
Kruskal-Wallis	Site:cumulative lesion development	30	3.69	0.158
Kruskal-Wallis	Site:total lesions at 46 weeks	30	4.06	0.131

Table S3. Results of Fisher's exact tests run to assess if site had any influence on the SCTLD status of a colony at each time point.

Test	Comparison	*n*	*p*-value
Fisher's Exact	Site:SCTLD status of colonies		
	At 3 weeks	32	0.242
	At 5 weeks	32	0.198
	At 9 weeks	32	0.139
	At 14 weeks	32	0.074
	At 23 weeks	32	0.224
	At 46 weeks	32	0.113

Table S4. Results of Kruskal-Wallis tests run to assess if the initial number of SCTLD lesions present on colonies was different between sites or treatment groups.

Test	Comparison	*n*	*H*	*p*-value
Kruskal-Wallis	Initial lesions present:site	32	0.466	0.792
Kruskal-Wallis	Initial lesions present:treatment	32	1.157	0.561

Table S5. Results of Kruskal-Wallis tests run to assess if initial number of SCTLD lesions present on colonies influenced it’s SCTLD status at each time point, first with all colonies grouped and then blocked by treatment. NAs represent cases when a comparison was unable to be made due to there being no difference in responses measured for that variable.

**Test**	**Comparison**	***n***	***H***	***p*-value**
Kruskal-Wallis	Initial lesions:SCTLD status of colony at 3 weeks			
	All treatments	32	5.949	0.429
	Amoxicillin only	11	0.833	0.659
	Chlorine only	11	2.667	0.615
	Untreated only	10	NA	NA
Kruskal-Wallis	Initial lesions:SCTLD status of colony at 5 weeks			
	All treatments	32	4.776	0.573
	Amoxicillin only	11	0.833	0.659
	Chlorine only	11	2.667	0.615
	Untreated only	10	NA	NA
Kruskal-Wallis	Initial lesions:SCTLD status of colony at 9 weeks			
	All treatments	32	3.663	0.722
	Amoxicillin only	11	0.179	0.915
	Chlorine only	11	NA	NA
	Untreated only	10	2.333	0.675
Kruskal-Wallis	Initial lesions:SCTLD status of colony at 14 weeks			
	All treatments	32	3.275	0.774
	Amoxicillin only	11	2.413	0.343
	Chlorine only	11	NA	NA
	Untreated only	10	1.285	0.864
Kruskal-Wallis	Initial lesions:SCTLD status of colony at 23 weeks			
	All treatments	32	6.035	0.419
	Amoxicillin only	11	2.667	0.264
	Chlorine only	11	NA	NA
	Untreated only	10	2.958	0.565
Kruskal-Wallis	Initial lesions:SCTLD status of colony at 46 weeks			
	All treatments	32	7.438	0.282
	Amoxicillin only	11	2.143	0.343
	Chlorine only	11	3.507	0.477
	Untreated only	10	3.583	0.465

Table S6. Results of Kruskal-Wallis tests run to assess if initial colony size was different across sites or treatment groups, and to determine if initial colony size influenced it's SCTLD status at 46 weeks, first with all colonies grouped and then blocked by treatment.

Test	Comparison	*n*	*H*	*p*-value
Kruskal-Wallis	Initial surface area:site	30	0.322	0.851
Kruskal-Wallis	Initial surface area:treatment	30	1.900	0.387
Kruskal-Wallis	Initial surface area:SCTLD status of colonies			
	All treatments	30	5.814	0.055
	Amoxicillin only	10	0.325	0.569
	Chlorine only	11	1.042	0.307
	Untreated only	9	3.278	0.194

Table S7. Results of Spearman's rank correlation analyses run to assess if there was a relationship between time and new or total lesions developed, first with all colonies grouped and then blocked by treatment. Significant *p*-values are bolded.

Test	Comparison	*n*	rho	*p*-value
Spearman's Rank Correlation	New lesions developed:time			
	All treatments	30	0.065	0.375
	Amoxicillin only	11	0.051	0.685
	Chlorine only	11	0.2	0.115
	Untreated only	8	-0.016	0.907
Spearman's Rank Correlation	Total lesions present:time			
	All treatments	30	-0.088	0.229
	Amoxicillin only	11	0.064	0.609
	Chlorine only	11	-0.033	0.791
	Untreated only	8	-0.19	0.157

Table S8. Results of Kruskal-Wallis tests run to assess if treatment influenced new lesions developed between each time point, or the cumulative number of new lesions developed over the entire course of the experiment.

Test	Comparison	*n*	*H*	*p*-value
Kruskal-Wallis	Treatment:new lesions developed			
	From 0−3 weeks	32	0.622	0.733
	From 3−5 weeks	32	0.848	0.655
	From 5−9 weeks	31	5.068	0.079
	From 9−14 weeks	31	1.902	0.386
	From 14−23 weeks	31	0.034	0.983
	From 23−46 weeks	30	3.5	0.174
Kruskal-Wallis	Treatment:cumulative new lesions developed	30	1.759	0.415
